# Value of preoperative controlling nutritional status score in prognosis of patients with high-risk factors for early-stage cervical cancer

**DOI:** 10.12669/pjms.40.1.7406

**Published:** 2024

**Authors:** FuYan Tan, RuMin Xia, Lanjuan Zeng, Huan Xie, Xiaolu Long, Caijiao Peng

**Affiliations:** 1 FuYan Tan, Department of Obstetric and Gynecologic, Hunan University of Medicine General Hospital, Huaihua 418000, Hunan, China; 2 RuMin Xia, Department of Obstetric and Gynecologic, Hunan University of Medicine General Hospital, Huaihua 418000, Hunan, China; 3 Lanjuan Zeng, Department of Obstetric and Gynecologic, Hunan University of Medicine General Hospital, Huaihua 418000, Hunan, China; 4 Huan Xie, Department of Obstetric and Gynecologic, Hunan University of Medicine General Hospital, Huaihua 418000, Hunan, China; 5 Xiaolu Long, Department of Obstetric and Gynecologic, Hunan University of Medicine General Hospital, Huaihua 418000, Hunan, China; 6 Caijiao Peng, Department of Obstetric and Gynecologic, Hunan University of Medicine General Hospital, Huaihua 418000, Hunan, China

**Keywords:** Cervical cancer, Preoperative controlling nutritional status, High-risk factor, Prognosis

## Abstract

**Objective:**

To investigate whether the preoperative controlling nutritional status (COUNT) score is a prognostic factor of patients with high-risk factors for early-stage cervical cancer after surgery and concurrent chemoradiotherapy (CCRT).

**Methods:**

This was a retrospective study. From July 2017 to March 2021, a total of 354 patients with histologically confirmed FIGO stage IB-IIA cervical cancer undergoing surgery and postoperative CCRT were included at Hunan University of Medicine General Hospital, China. According to receiver operating characteristic (ROC) curve analysis, the patients were divided into a low CONUT score (< 3) group and a high CONUT score (≥ 3) group. Overall survival (OS) was used as the primary outcome measure and disease-free survival (DFS) as the secondary outcome measure.

**Results:**

Among the 354 patients, 239(67.5%) were included in the low CONUT score group and 115 (32.5%) in the high CONUT score group. The 3, 5 and 10-year OS rates in the low CONUT score group and high CONUT score group were respectively presenting statistically significant differences (*p<*0.001). The 3, 5 and 10-year DFS rates in the low CONUT score group and in the high CONUT score group were respectively with statistically significant differences (*p<*0.001). Multivariate Cox regression analysis showed that CONUT score, histological type, PNI and lymph node metastasis were all independent predictors for OS and DFS (all *p<*0.05).

**Conclusion:**

High preoperative CONUT score indicates poor prognosis of patients with high-risk factors for early-stage cervical cancer after surgery and postoperative CCRT. In clinical practice, consolidation chemotherapy is recommended for patients with high CONUT scores.

## INTRODUCTION

Cervical cancer is the fourth most common cancer among women. Patients with early-stage cervical cancer (FIGO stage IB-IIA) are usually treated with radical hysterectomy and pelvic lymph node dissection.^1^ The clinicopathological parameters such as postoperative lymph node metastasis, parametrial invasion and positive surgical margin are the high-risk factors for tumor recurrence. Postoperative concurrent chemoradiotherapy (CCRT) is recommended for patients with high-risk factors.^2^ Although the survival rate of patients with high-risk factors for early-stage cervical cancer after postoperative CCRT is high, some patients present poor prognosis, mainly distant metastasis. It has been previously shown that consolidation chemotherapy can be considered for some patients after primary treatment to eradicate occult distant metastasis.^3^ However, how to screen patients with poor prognoses for consolidation therapy is a challenge faced by clinicians.

Studies have shown that nutritional status and immune status are related to tumor progression and prognosis.^4^ For example, neutrophil-lymphocyte ratio (NLR), platelet-lymphocyte ratio (PLR), prognostic nutritional index (PNI), systemic immune-inflammatory index (SII) and nutritional risk index can be used to evaluate the prognosis of patients with tumors.^4^,^5^ The preoperative controlling nutritional status (CONUT) score is a comprehensive index calculated from serum albumin level, total cholesterol level and total peripheral blood lymphocyte count, which can be used as a nutritional index to evaluate nutritional status.^6^ It has been shown that the ONUT score is related to the prognosis in esophageal cancer^7^, gastric cancer^8^ and liver cancer.^9^ However, in early-stage cervical cancer, the relationship between CONUT score and prognosis is still unclear. On this basis, this study aims to explore the predictive value of the CONUT score in the prognosis of patients with high-risk factors for early-stage cervical cancer after postoperative CCRT.

## METHODS

This was a retrospective study, from July 2017 to March 2021, patients with histologically confirmed FIGO stage IB-IIA (2009) cervical cancer undergoing treatment at Hunan University of Medicine General Hospital, China were included. Patient data were collected from the electronic medical record system of our hospital, including baseline characteristics, medical history, physical examination and laboratory examination.

### Ethical Approval

The study was approved by the Institutional Ethics Committee of Hunan University of Medicine General Hospital (No.: KY-2022062915; date: June 29, 2022), and written informed consent was obtained from all participants.

### Inclusion criteria:

- FIGO stage IB-IIA.
- radical hysterectomy and pelvic lymph node dissection and/or paraaortic lymph node sampling.
- histologically confirmed high-risk factors (lymph node metastasis, parametrial invasion and positive surgical margin).
- postoperative CCRT.

### Exclusion criteria

- Patients with preoperative urinary system infections, bladder tumors, urinary calculus and other urinary system diseases.
- Patients with dysfunction of the heart, kidney, liver and other important organs.
- Patients with preoperative radiotherapy or postoperative complications.

### Treatment plan

All the patients received intensity-modulated radiotherapy (IMRT), which included conventional fixed-field intensity-modulated radiotherapy and helical tomotherapy (HT). The clinical target volume (CTV) consisted of the regional lymph node area (obturators, internal and external iliac and common iliac lymph node area) and upper vagina. During external irradiation, the prescribed dose of 45~50.4 Gy was given to the planned target volume (PTV), 25-28 times. Conical beam CT was performed weekly for patients receiving conventional IMRT, and airborne high-voltage CT daily for patients receiving HT before treatment. Concurrent chemotherapy was carried out with 40 mg/m^2^ cisplatin, six cycles per week. For some patients with renal complications, 60 mg/m^2^ paclitaxel was administered weekly.

### CONUT score and other clinical parameters

**Fig.1:**
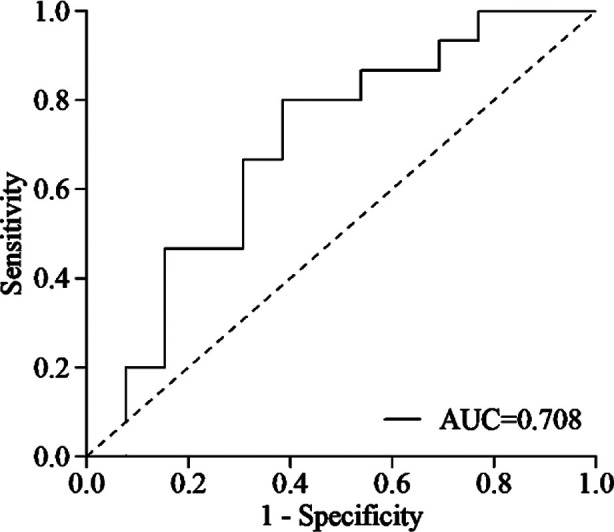
Receiver operating characteristic curve for CONUT score.

The albumin level, total lymphocyte count and total cholesterol level of each patient within one week before surgery were collected, and the COUNT score was calculated with the method mentioned in the literature.^9^ In our study, the optimal cut-off of the CONUT score was determined as three based on the receiver operating characteristic (ROC) curve (Fig.1). CONUT score ≥ 3 was defined as a high CONUT score group, and CONUT score < 3 as a low CONUT score group. According to the recommendation of the measurement kit, the optimal cut-off of SCC-Ag was 2.0 ng/mL. PNI was calculated by albumin level (g/L) × total lymphocyte count × 10^9^/L.

Follow-up: Starting from one month after the initial treatment, follow-up was conducted every three months for two years, every six months in the later 3-5 years, and once a year after five years. Routine follow-up evaluation included gynecological examination, blood cell count, squamous cell carcinoma detection with silver nanoclusters, abdominal and pelvic contrast-enhanced MRI, and chest contrast-enhanced CT. In the case of suspected recurrence, PET/CT and biopsy were performed. The patients were followed up until June 2021 or death.

### Statistical Analysis

The data were statistically analyzed using SPSS 19.0. Local recurrence was defined as recurrence in the irradiation field and distant recurrence as recurrence outside the irradiation field. The main outcome measure was overall survival (OS), which was defined as the time interval from the beginning of surgery to death from any cause. The secondary outcome measure was disease-free survival (DFS), which was defined as the time interval from the beginning of surgery to disease recurrence or death from any cause.

The measurement data were expressed as mean ± standard deviation, and their comparisons between the groups were performed using the independent sample t-test. The categorical data were described by the number of cases (percentage), and compared between the groups using the chi-square test or Fisher’s exact test. The optimal cut-off of the COUNT score was analyzed by the ROC curve with OS as the dependent variable. The Kaplan-Meier curve was used to calculate the survival rate and log-rank test for comparison between groups. Multivariate Cox proportional hazards regression analysis was carried out for OS and DFS to determine their independent influencing factors. The power of test / confidence interval was 95%. All tests were bilateral, and *p<*0.05 was considered statistically significant.

## RESULTS

From July 2017 to March 2021, a total of 615 patients with histologically confirmed FIGO stage IB-IIA cervical cancer were screened out. After excluding 130 patients receiving neoadjuvant chemotherapy, 72 patients receiving surgery without adjuvant therapy and 59 patients receiving radiotherapy only, 354 patients receiving CCRT after surgery were finally included for analysis. The median age of the 354 patients was 58.5 years (range: 32~78 years). BMI, FIGO stage, LVSI, lymph node metastasis and PNI showed no statistically significant differences between the two groups (all *p<*0.05). Table-I.

**Table-I:** Baseline characteristics of patients with early-stage cervicalcancer classified by CONUT score.

Characteristics	Low COUNT score (*n* = 239)	High COUNT score (*n* = 115)	χ^2^	*P*
Age (year)	57.2 ± 11.3	58.1 ± 12.3	0.682	0.496
***BMI (kg/m^2^)***			3.609	0.049
< 18.5	146 (61.1%)	58 (50.4%)		
≥ 18.5	93 (38.9%)	57 (49.6%)		
***FIGO stage***			8.725	0.003
IB	199 (83.3%)	80 (69.6%)		
IIA	40 (16.7%)	35 (30.4%)		
***Hemoglobin (g/L)***			1.383	0.240
≥ 110	221 (92.5%)	102 (88.7%)		
< 110	18 (7.5%)	13 (11.3%)		
***Histological type***			2.892	0.236
SCC	180 (75.3%)	77 (67.0%)		
AD	47 (19.7%)	29 (25.2%)		
ADSQ	12 (5.0%)	9 (7.8%)		
***DSI***			0.936	0.333
≥ 50%	210 (87.9%)	105 (91.3%)		
< 50%	29 (12.1%)	10 (8.7%)		
***LVSI***			38.526	< 0.001
+	182 (76.2%)	49 (42.6%)		
-	57 (23.8%)	66 (57.4%)		
***Primary tumor size (cm)***			3.358	0.067
≥ 4	102 (42.7%)	61 (53.0%)		
< 4	137 (57.3%)	54 (47.0%)		
***Lymph node metastasis***			10.623	0.001
+	197 (82.4%)	77 (67.0%)		
-	42 (17.6%)	38 (33.0%)		
***Parametrial invasion***			2.531	0.112
+	150 (62.8%)	62 (53.9%)		
-	89 (37.2%)	53 (46.1%)		
***Positive surgical margin***			< 0.001	> 0.999
+	7 (2.9%)	4 (3.5%)		
-	232 (97.1%)	111 (96.5%)		
***SCC-Ag***			0.075	0.784
Normal	53 (22.2%)	27 (23.5%)		
Elevated	186 (77.8%)	88 (76.5%)		
***PNI***			101.721	< 0.001
> 48.5	239 (100.0%)	72 (62.6%)		
≤ 48.5	0 (0.0%)	43 (37.4%)		
***HDR brachytherapy***			3.198	0.074
Yes	115 (48.1%)	67 (58.3%)		
No	124 (51.9%)	48 (41.7%)		
Concurrent chemotherapy			0.847	0.357
Cisplatin	228 (95.4%)	107 (93.0%)		
Paclitaxel	11 (4.6%)	8 (7.0%)		

CONUT, controlling nutritional status; FIGO, International Federation of Gynecology and Obstetrics; BMI, body mass index; DSI, deep stromal invasion; LVSI, lymphovascular space invasion; SCC-Ag, squamous cell carcinoma antigen; SCC, squamous cell carcinoma; AD, adenocarcinoma; ADSQ, adenosquamous carcinoma; PNI, prognostic nutritional index; HDR, high-dose-rate.

**Fig.2:**
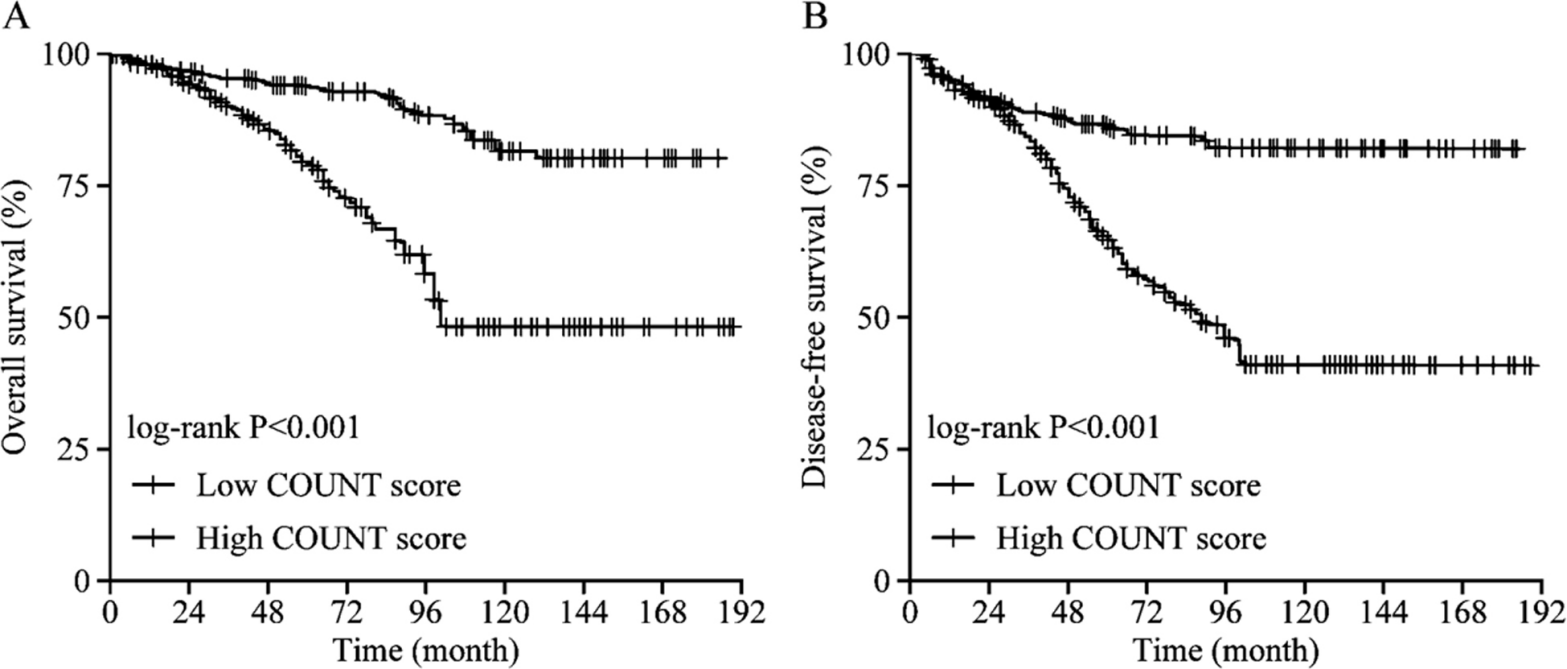
Kaplan-Meier curves of overall survival (A) and disease-free survival (B) according to COUNT score.

The median follow-up time was 51.3 months (range: 3.5~197.4 months). A total of 322 (90.1%) patients survived, and 68 (19.2%) patients were clinically or radiologically proven recurrent at the last follow-up. Kaplan-Meier analysis showed that the three, five and ten years OS rates in the low CONUT score group were 94.4%, 93.1% and 81.1%, while those in the high CONUT score group were 89.4%, 79.3% and 47.4%, respectively, presenting statistically significant differences (*p<*0.001), as seen in Figure. 2A. The 3, 5 and 10-years DFS rates were 88.0%, 84.5% and 79.7% in the low CONUT score group, and 81.3%, 65.0% and 39.3% in the high CONUT score group, respectively, with statistically significant differences (*p<*0.001) (Figure.2B).

Univariate Cox regression analysis showed that histological type, lymph node metastasis, SCC-Ag, PNI and CONUT score were correlated with OS (all *p<*0.05). Multivariate Cox regression analysis revealed that CONUT score, histological type and lymph node metastasis were all independent predictors of OS (all *p<*0.05).Table-II

**Table-II:** Univariable and multivariable Cox analyses of factors correlated with overall survival for patients with early-stage cervical cancer.

Variables	Univariable		Multivariable	

	HR (95%CI)	P	HR (95%CI)	P
***Age (year)***				
< 60	1.00			
≥ 60	1.81 (0.98-3.32)	0.056		
***BMI (kg/m^2^)***				
< 18.5	1.00			
≥ 18.5	1.11 (0.92-1.33)	0.273		
***FIGO stage***				
IB	1.00			
IIA	1.43 (0.94-2.17)	0.094		
***Hemoglobin (g/L)***				
≥ 110	1.00			
< 110	1.19 (0.85-1.68)	0.310		
***Histological type***				
SCC	1.00		1.00	
Non-SCC	0.79 (0.75-0.83)	< 0.001	0.71 (0.72-0.85)	< 0.001
***Primary tumor size (cm)***				
≥ 4	1.00			
< 4	2.38 (0.95-5.94)	0.064		
***Lymph node metastasis***				
-	1.00		1.00	
+	2.06 (1.06-4.00)	0.032	2.35 (1.19-4.66)	0.014
***Parametrial invasion***				
-	1.00			
+	1.79 (0.94-3.38)	0.074		
***Positive surgical margin***				
-	1.00			
+	1.48 (0.90-2.45)	0.123		
***SCC-Ag***				
Normal	1.00		1.00	
Elevated	2.26 (1.11-4.58)	0.024	1.86 (0.98-3.50)	0.056
***PNI***				
> 48.5	1.00		1.00	
≤ 48.5	2.15 (1.16-4.00)	0.015	2.55 (1.13-5.74)	0.024
***HDR brachytherapy***				
No	1.00			
Yes	1.53 (0.86-2.74)	0.148		
***COUNT***				
< 3	1.00		1.00	
≥ 3	1.54 (1.15-2.07)	0.004	1.29 (1.05-1.59)	0.014

Univariate Cox regression analysis presented that lymph node metastasis, histological type, SCC-Ag, PNI and CONUT score were correlated with DFS (all *p<*0.05). Multivariate Cox regression analysis demonstrated that lymph node metastasis, histological type, PNI and CONUT score were independent predictors of DFS (all *p<*0.05) Table-III

**Table-III:** Univariable and multivariable Cox analyses of factors correlated with disease-free survival for patients with early-stage cervical cancer.

Variables	Univariable		Multivariable	

	HR (95%CI)	P	HR (95%CI)	P
***Age (year)***				
< 60	1.00			
≥ 60	1.72 (0.89-3.33)	0.109		
***BMI (kg/m^2^)***				
< 18.5	1.00			
≥ 18.5	1.11 (0.88-1.40)	0.390		
***FIGO stage***				
IB	1.00			
IIA	1.43 (0.84-2.42)	0.184		
***Hemoglobin (g/L)***				
≥ 110	1.00			
< 110	1.68 (0.96-2.93)	0.068		
***Histological type***				
SCC	1.00			
Non-SCC	0.43 (0.13-0.69)	0.011	0.44 (0.12-0.70)	0.012
***Primary tumor size (cm)***				
≥ 4	1.00			
< 4	2.51 (0.85-7.39)	0.095		
***Lymph node metastasis***				
-	1.00		1.00	
+	2.55 (1.21-5.36)	0.014	2.06 (1.20-3.54)	0.009
***Parametrial invasion***				
-	1.00			
+	1.86 (0.98-3.52)	0.058		
***Positive surgical margin***				
-	1.00			
+	1.35 (0.88-2.06)	0.172		
***SCC-Ag***				
Normal	1.00		1.00	
Elevated	1.67 (1.18-2.35)	0.004	1.55 (0.87-2.77)	0.135
***PNI***				
> 48.5	1.00		1.00	
≤ 48.5	2.35 (1.06-5.23)	0.036	1.79 (1.10-2.91)	0.019
***HDR brachytherapy***				
No	1.00			
Yes	1.72 (0.83-3.55)	0.142		
***COUNT***				
< 3	1.00		1.00	
≥ 3	2.26 (1.11-4.58)	0.025	1.53 (1.14-2.06)	0.004

## DISCUSSION

Our study found that the preoperative CONUT score was an independent prognostic factor of patients with high-risk factors for early-stage cervical cancer after surgery and postoperative CCRT. CONUT score, as a method to evaluate immune and nutritional status, can predict the prognosis of multiple solid tumors.^9^-^11^ However, the prognostic significance of the CONUT score in patients with high-risk factors for early-stage cervical cancer has not been clarified. This study showed that the CONUT score had strong correlations with BMI, FIGO stage, LVSI, lymph node metastasis and PNI, and was an independent prognostic factor of DFS and OS. Patients with higher CONUT scores presented poorer DFS and OS.

In malignant tumors, nutritional and immune status are related to prognosis.^5^,^12^,^13^ Haraga et al.^4^ have reported that PNI, as an immune and nutritional index, can predict the poor prognosis of patients with cervical cancer receiving CCRT. In our study, it was found that compared with PNI, the CONUT score might be more effective in evaluating prognosis, which may be related to its emphasis on total lymphocytes. In addition, compared with PNI, the CONUT score further evaluates the role of total cholesterol concentration. Therefore, this study suggests that the CONUT score may be a more comprehensive predictor of patients with high-risk factors for early-stage cervical cancer. A previous study has also found that the CONUT score is independently correlated with DFS and OS.^14^ Moreover, Iseki et al.^10^ have reported that the preoperative CONUT score is an effective independent predictor of the clinical outcome of patients with colorectal cancer, and has higher prediction accuracy than PNI.

CONUT score is composed of three variables including serum albumin level, total cholesterol level and total lymphocyte count. The role of these variables can clarify the predictive value of preoperative CONUT score for the prognosis of patients with high-risk factors for early stage cervical cancer after surgery and postoperative CCRT. Serum albumin is closely related to the degree of malnutrition and the prognosis of patients with gastric cancer.^15^ Low serum albumin level is related to the increase of tumor-associated inflammatory reactions, which may be caused by the decrease in albumin production by hepatocytes due to the release of inflammatory cytokines by tumor cells.^16^ Additionally, serum albumin can bind to and activate prostaglandin E2 (PGE2), which down-regulates macrophage-derived tumor necrosis factor-α (TNF-α) and promotes immunosuppression.^17^

A previous study^18^ has found that the low serum cholesterol level in patients with hepatocellular carcinoma is related to poor prognosis, and the proliferation and growth of tumor cells may lead to a reduction in serum cholesterol level.^19^ Lymphocytes are an important component of host immunity, and the decrease in lymphocytes can lead to tumor immunological disorders.^20^ The weakened immune surveillance is caused by low lymphocyte count in combination with other tumor-promoting inflammatory factors, which provides a good condition for the growth and progression of tumor cells. Combined with these three parameters, the CONUT score can be included in different nutritional stages to further improve the accuracy in evaluating immune status.

### Limitations

Firstly, this study is a retrospective study, and patient enrollment may have potential selection bias. Secondly, the nutritional support after postoperative CCRT in this study was not followed up in detail, so the CONUT score after treatment can not be obtained and its significance can not be recognized.

## CONCLUSION

Our study reveals that a high preoperative CONUT score indicates poor prognosis of patients with high-risk factors for early-stage cervical cancer after surgery and postoperative CCRT. CONUT score may be an effective index for these patients to use consolidation chemotherapy and predict clinical outcomes.

### Authors’ Contributions:

**FT**and **RX:** Carried out the studies, participated in collecting data, drafted the manuscript, are responsible and accountable for the accuracy and integrity of the work.

**LZ** and **HX:** Performed the statistical analysis and participated in its design.

**XL** and **CP:** Participated in acquisition, analysis, or interpretation of data and draft the manuscript. All authors read and approved the final manuscript.
